# Integrative analyses of biomarkers and pathways for heart failure

**DOI:** 10.1186/s12920-022-01221-z

**Published:** 2022-03-27

**Authors:** Shaowei Fan, Yuanhui Hu

**Affiliations:** Guang’anmen Hospital, China Academy of Chinese Medical Sciences, Beijing, China

**Keywords:** Potential key genes, Heart failure, Bioinformatics, Gene expression synthesis, Biomarkers

## Abstract

**Background:**

Heart failure (HF) is the most common potential cause of death, causing a huge health and economic burden all over the world. So far, some impressive progress has been made in the study of pathogenesis. However, the underlying molecular mechanisms leading to this disease remain to be fully elucidated.

**Methods:**

The microarray data sets of GSE76701, GSE21610 and GSE8331 were retrieved from the gene expression comprehensive database (GEO). After merging all microarray data and adjusting batch effects, differentially expressed genes (DEG) were determined. Functional enrichment analysis was performed based on Gene Ontology (GO) resources, Kyoto Encyclopedia of Genes and Genomes (KEGG) resources, gene set enrichment analysis (GSEA), response pathway database and Disease Ontology (DO). Protein protein interaction (PPI) network was constructed using string database. Combined with the above important bioinformatics information, the potential key genes were selected. The comparative toxicological genomics database (CTD) is used to explore the interaction between potential key genes and HF.

**Results:**

We identified 38 patients with heart failure and 16 normal controls. There were 315 DEGs among HF samples, including 278 up-regulated genes and 37 down-regulated genes. Pathway enrichment analysis showed that most DEGs were significantly enriched in BMP signal pathway, transmembrane receptor protein serine/threonine kinase signal pathway, extracellular matrix, basement membrane, glycosaminoglycan binding, sulfur compound binding and so on. Similarly, GSEA enrichment analysis showed that DEGs were mainly enriched in extracellular matrix and extracellular matrix related proteins. BBS9, CHRD, BMP4, MYH6, NPPA and CCL5 are central genes in PPI networks and modules.

**Conclusions:**

The enrichment pathway of DEGs and GO may reveal the molecular mechanism of HF. Among them, target genes EIF1AY, RPS4Y1, USP9Y, KDM5D, DDX3Y, NPPA, HBB, TSIX, LOC28556 and XIST are expected to become new targets for heart failure. Our findings provide potential biomarkers or therapeutic targets for the further study of heart failure and contribute to the development of advanced prediction, diagnosis and treatment strategies.

## Background

Cardiovascular disease is one of the main causes of human death, including coronary heart disease, hypertension, congenital heart disease, heart failure and other heart related diseases, as well as systemic vascular system related diseases such as atherosclerosis and lower extremity deep venous thrombosis. Among them, heart failure is the most common cardiovascular disease in clinic. It is estimated that about 64.3 million people worldwide suffer from heart failure [1]. As everyone knows, heart failure often follows a variety of other diseases, such as coronary heart disease, hypertension, diabetes, etc. these diseases are characterized by an obvious age-dependent dependency. That is, the higher the risk factor for age, the greater the risk of diseases. However, research shows that the burden of heart failure in young people may be increasing, which means that more young people will enter the ranks of heart failure, and its prevalence tends to be younger [2].

In terms of the mechanism of heart failure, myocardial hypertrophy is the key to the occurrence and development of heart failure, but the exact reason for the transformation of myocardial hypertrophy into heart failure is not clear. In this process, it may play a role in the pathological increase of the circulating level of vasoactive substances (such as angiotensin II, catecholamine, endothelin, etc.), and the increase of the content of vasoactive substances will stimulate the myocardium and eventually lead to myocardial hypertrophy by stimulating their respective signal transduction pathways [3]. When hypertrophic cardiomyocytes are exposed to the environment with increased content of vasoactive substances for a long time, it will lead to subcellular defects, protease activation, metabolic disorders, abnormal calcium regulation, etc., which will increase the dysfunction of heart function, thus aggravating the occurrence and development process of heart failure [4]. Therefore, the cytokines and other molecular mechanisms involved in the occurrence and development of heart failure still need to be further studied, and its specific mechanism needs to be further proved at the molecular level [5].

Nowadays, the treatment cost of patients with heart failure has always been a large expenditure for any country. Therefore, early diagnosis of heart failure is of great significance for early intervention and treatment, which is equally important for individuals and countries. At present, the diagnosis of heart failure is mainly achieved by the results of BNP, NT-proBNP, echocardiography and other clinical symptoms, such as fatigue, dyspnea, low body position fluid retention. BNP and NT-proBNP are gold standard biomarkers for the diagnosis and prognosis of heart failure. Therefore, obtaining objective, accurate, reliable, noninvasive and biologically meaningful biomarkers of heart failure will greatly optimize the diagnosis, monitoring, treatment and prognosis of heart failure, which will also become the research focus for a long time in the future.

With the rapid development of high-throughput technology, various studies related to the pathophysiological process of heart failure continue to deepen, and more and more new biomarkers have been found, such as middle regional preatrial natriuretic peptide (MR-proANP), middle regional adrenomedullin (MR-proADM), highly sensitive troponin, soluble ST2 (sST2) [6], growth differentiation factor (GDF)-15, Galectin-3 [7, 8], copeptin [8, 9], Cystatin C (Cys-C) [10] and Sirtuin (SIRT) [11], which have partially shown the potential to determine the diagnosis and prognosis of heart failure, but they are still insufficient in clinical evidence. In neurohumoral activities, pathological changes have taken place in the expression level of relevant biomarkers, signal molecules, cytokines and other substances often in the early stage of symptoms or even when there are no symptoms. Therefore, the cytokines and other molecular mechanisms involved in the occurrence and development of heart failure need to be further studied, in order to explore more biomarkers related to heart failure and improve their relevant clinical evidence, so as to improve the early diagnosis and prognosis management of heart failure and bring well-being to patients.

Heart failure is a major health problem in the world. It is necessary to explore the potential biomarkers and molecular mechanisms related to the occurrence and development of heart failure, so as to provide more targeted and effective treatment strategies for patients and bring benefits. At present, high-throughput technology has developed rapidly and is widely used in various fields. Integrated bioinformatics analysis is expected to become a key technology to clarify the etiology, pathogenesis and treatment of heart failure, which will benefit mankind in terms of human, material and financial resources.

In this study, we analyzed three mRNA expression profiles (GSE76701, GSE21610 and GSE8331) of the same platform (GPL570) downloaded from GEO Database (https://www.ncbi.nlm.nih.gov/geo/) to determine the possible DEGs in the occurrence and development of heart failure, and analyzed their expression, function and interaction, so as to provide reference for exploring biomarkers or therapeutic targets of heart failure.

## Methods

**Fig. 1:**
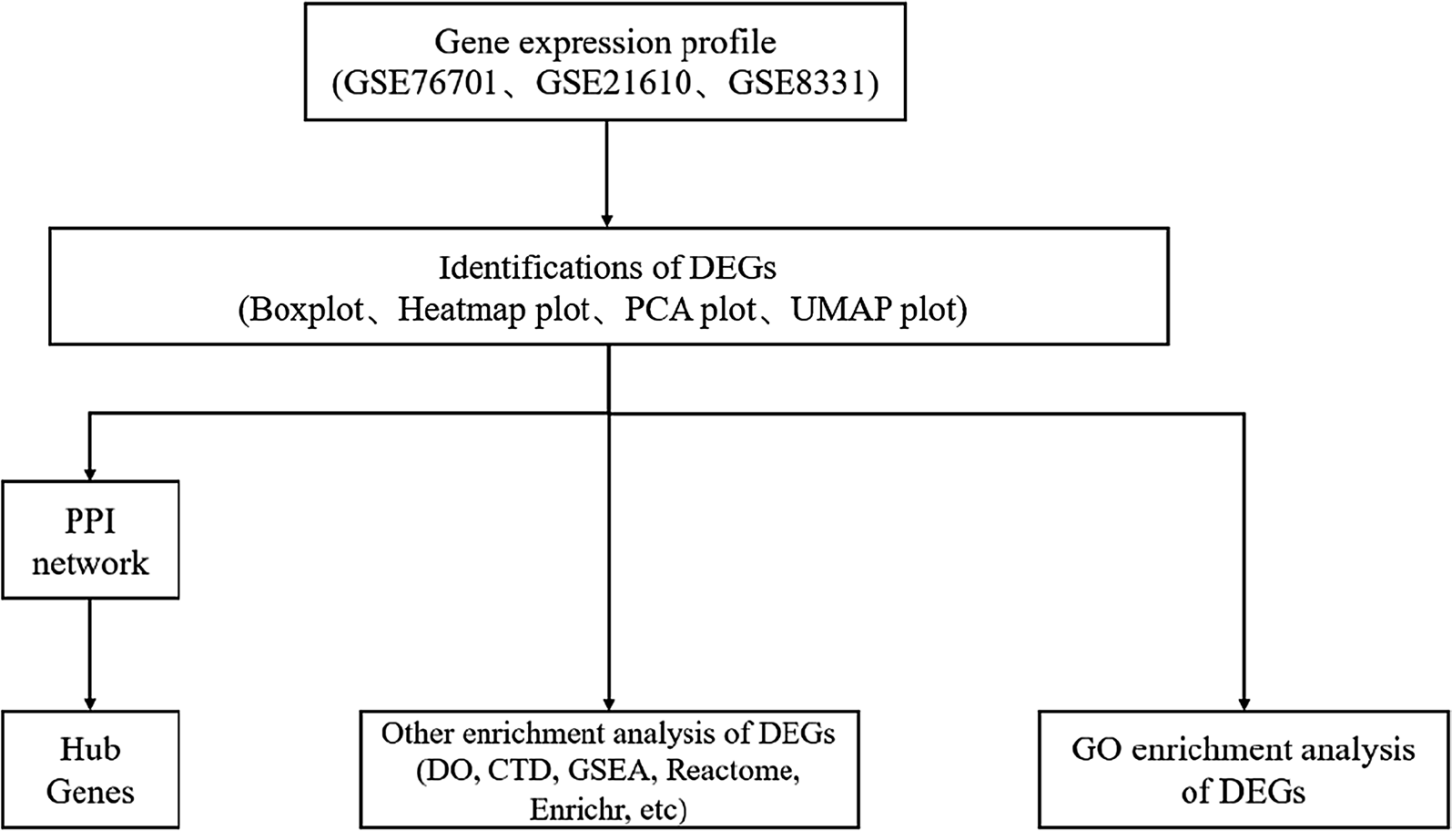
Study design (flow diagram of study). *PPI* Protein–protein interaction; *DO* Disease Oncology; CTD The Comparative Toxicogenomics Database; *GSEA* Gene Set Enrichment Analysis; *GO* Gene *Oncology*

Figure 1 clearly illustrates the flow chart of materials and methods. In this study, we integrated three datasets from GEO Database: GSE76701, GSE21610 and GSE8331. The characteristics of differential genes were displayed by Box plot, Heatmap plot, PCA plot and UMAP plot. Then, the construction of PPI network, the display of Hub gene, GO enrichment analysis and other enrichment analysis including DO, CTD, GSEA, Reactome and Enrichr were listed.

### Heart failure dataset

Download the heart failure dataset original files of three registered microarray datasets from NCBI GEO Database, including GSE76701, GSE21610 and GSE8331 (Table 1). All these datasets are from the microarray platform of Affymetrix Human Genome U133 Plus 2.0 Array [HG-U133_Plus_2]. In each dataset, human myocardial samples were selected only from HF and normal EF subjects, and finally 38 HF and 16 normal EF group samples were included for subsequent analysis.

**Table 1:** Characteristics of datasets in this study

GSE series	Platform	Total	HF	NG	Organism	Country	Contributors
GSE76701	GPL570	8	4	4	Myocardium	America	Kim EH, et al
GSE21610	GPL570	38	30	8	Myocardium	Germany	Schwientek P, et al
GSE8331	GPL570	8	4	4	Myocardium	Japan	Mano H

GSE, Gene Expression Omnibus; HF, Heart Failure; NG, Normal Group

### Data preprocessing

The GSE76701 dataset contains 4 human HF samples and 4 human health samples, the GSE21610 dataset contains 30 human HF samples and 8 human health samples, and the GSE8331 dataset contains 4 human HF samples and 4 human health samples. A series of matrix text files for the dataset have been obtained. Subsequently, the limma R software package was used for background correction, quartile standardization and probe summary [12–15]. See Table 1 for details.

### Identification of DEGs between HF and healthy samples

In this study, we used the limma R package (version 3.6.3; https://www.r-project.org/) [12]. The DEGs between HF samples and healthy samples were determined by the threshold standard of |log2 (FC)|> 1 and *p*.adj < 0.05.

### Function and pathway enrichment analysis of DEGs

Gene Ontology (GO) resources (http://geneontology.org/, Accessed 20 Oct 2021) is a bioinformatics tool that provides a framework and a set of concepts to describe the function of all biological gene products [16]. Kyoto Encyclopedia of Genes and Genomes (KEGG) (https://www.kegg.jp/) is a database resource integrated the information of genomes, biological pathways, diseases and chemicals [17–19]. Reactome pathway database (https://reactome.org/) is a path annotation database that collects human biological paths and processes [20]. Enrichr (https://maayanlab.cloud/Enrichr/, Accessed 20 Oct 2021) is an online data processor [21–23]. Disease Ontology (DO) (http://disease-ontology.org, Accessed 20 Oct 2021), represents a comprehensive knowledge base of 8043 genetic, developmental and acquired human diseases [24]. Gene Set Enrichment Analysis (GSEA) (http://software.broadinstitute.org/gsea/index.jsp, Accessed 20 Oct 2021) is a computational method for interpreting gene expression data based on molecular signature database [25, 26]. Before enrichment analysis, the gene symbol code is converted into Entrez ID using the human genome annotation package "org. HS. eg.db". In order to better understand the biological functions and characteristics, the enrichment analysis is carried out with R software, using "Clusterprofiler" KEGG and GO enrichment analysis package, reactor pathway analysis "Reactomepa" package and do enrichment analysis "DOSE" package. The "GOplot" and "ggplot2" packages of R software are used for visual mapping. The relevant go biological function map is considered to be significantly rich if it meets the *p*. adjust value < 0.05 and Q value < 0.05. For the important paths related to heart failure, the significance level, nominal *p* value and false discovery rate The cutoff value of (FDR) Q value is 0.05. For GSEA enrichment analysis, FDR Q value < 0.25 and *p*.adjust value < 0.05 are used as screening indexes.

### Protein–protein interaction (PPI) network and potential key gene analysis

String database (http://string-db.org/, Accessed 20 Oct 2021) is used to construct PPI network to reveal the general organization principle of functional cell system and predict protein–protein interaction [27]. Through molecular complex detection (MCODE) of Cytoscape, the results of PPI network are modularized analyzed and visualized. Default parameters (degree cutoff) ≥ 2, node score cut-off ≥ 2, K nucleus ≥ 2, maximum depth = 100). In order to select potential key genes, we synthesized the above important bioinformatics information for subsequent analysis. If DEG meets the inclusion criteria (adjusted *p* value < 0.05 and |log2 FC|≥ 1), it is considered as potential key genes. In addition, genes with connectivity greater than 5 in PPI network are also included.

### Identification of potential key genes associated with heart failure

The Comparative Toxicogenomics Database (CTD, http://ctdbase.org/, Accessed 20 Oct 2021) synthesize information, including chemical gene/protein interactions, chemical diseases and gene disease relationships, to develop hypotheses related to disease mechanisms [28]. Use data in CTD to analyze the association between potential key genes and the risk of heart failure, atrial fibrillation, hypertension and sudden cardiac death.

## Results

### Dataset evaluation

**Fig. 2:**
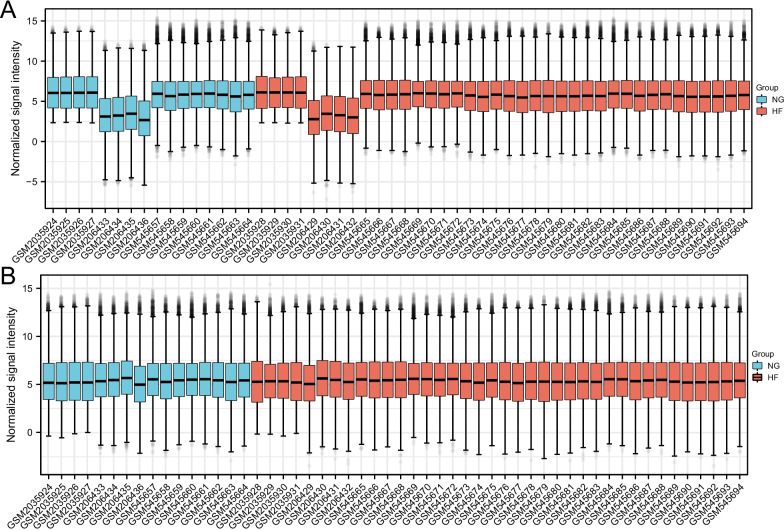
Boxplots of gene expressions before and after standardization for 3 selected GEO Database. (**A** Before standardization; **B** After standardization. NG: Normal Group; HF: Heart Failure Group)

**Fig. 3:**
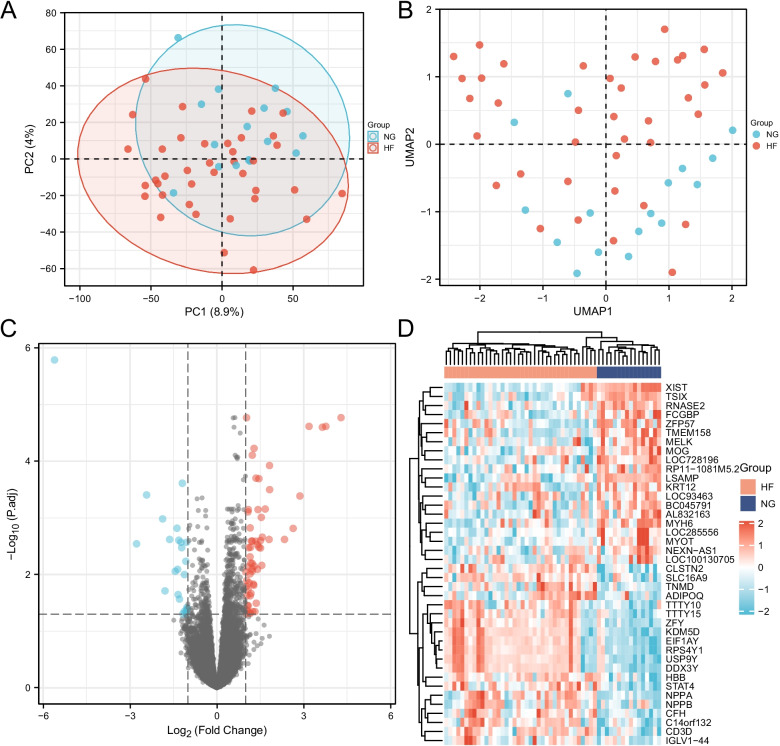
The PCA plot, UMAP plot, Volcano plot and Heatmap plot of gene expressions for 3 selected GEO Database with a screening criteria of |log2 FC|≥ 1 and adjust *P* value < 0.05. (**A** PCA plot; **B** UMAP plot; **C** Volcano plot; **D** Heatmap plot. PCA: Principal Component Analysis; UMAP: Uniform Manifold Approximation and Projection; NG: Normal Group; HF: Heart Failure Group)

The gene expression level of the combined GEO series with adjusted batch effect is standardized, and the results before and after standardization are shown in Fig. 2. Probes corresponding to 21,655 genes in GSE76701, GSE21610 and GSE8331 datasets were identified, and DEGs of heart failure were confirmed. The total number of filtered molecules was 21,655, of which 85 IDs met the threshold of |log2 (FC)|≥ 1 & *p*.adj < 0.05. Under this threshold, 60 were highly expressed in HF group and 25 in normal group; 22 IDs met the threshold of |log2 (FC)|≥ 1.5 & *p*.adj < 0.05. Under this threshold, 16 IDs were highly expressed in HF group and 6 IDS were highly expressed in normal group; There are 10 IDs that meet the threshold of |log2 (FC)|≥ 2 & *p*.adj < 0.05. Under this threshold, there are 7 highly expressed IDs in HF group (EIF1AY, RPS4Y1, USP9Y, KDM5D, DDX3Y, NPPA and HBB) and 3 highly expressed IDS in normal group (TSIX, LOC28556 and XIST). See Table 2 for details. Figure 3 shows the Heatmap plot, Volcano plot, PAC plot and UMAP plot.

**Table 2:** The DEGs of merged data set with the use of criteria of adjust *P* value < 0.05 and |logFC|≥ 2

Gene	LogFC	AveExpr	t	*P* value	Adjust *P* value	B
XIST	− 5.60291	6.415566	− 8.05301	7.55E−11	1.64E−06	14.17664
EIF1AY	4.290193	7.905949	7.068635	3.03E−09	1.71E−05	10.80004
RPS4Y1	3.765519	8.68866	6.835021	7.29E−09	2.43E−05	9.993851
USP9Y	3.646357	5.569263	6.73868	1.05E−08	2.52E−05	9.661358
KDM5D	3.185601	6.035742	6.814739	7.87E−09	2.43E−05	9.923851
DDX3Y	2.869435	6.737341	5.715082	4.73E−07	0.000409	6.15164
LOC285556	− 2.77444	4.659843	− 4.77551	1.39E−05	0.002863	3.038569
NPPA	2.635568	10.70313	5.101512	4.38E−06	0.00153	4.099873
TSIX	− 2.42784	4.207833	− 5.73785	4.35E−07	0.000392	6.228833
HBB	2.336796	10.47484	4.92105	8.32E−06	0.002397	3.509395

LogFC, log Fold Change; AveExpr, Average Expression

### Functional enrichment analysis of DEGs

#### GO enrichment analysis

**Fig. 4:**
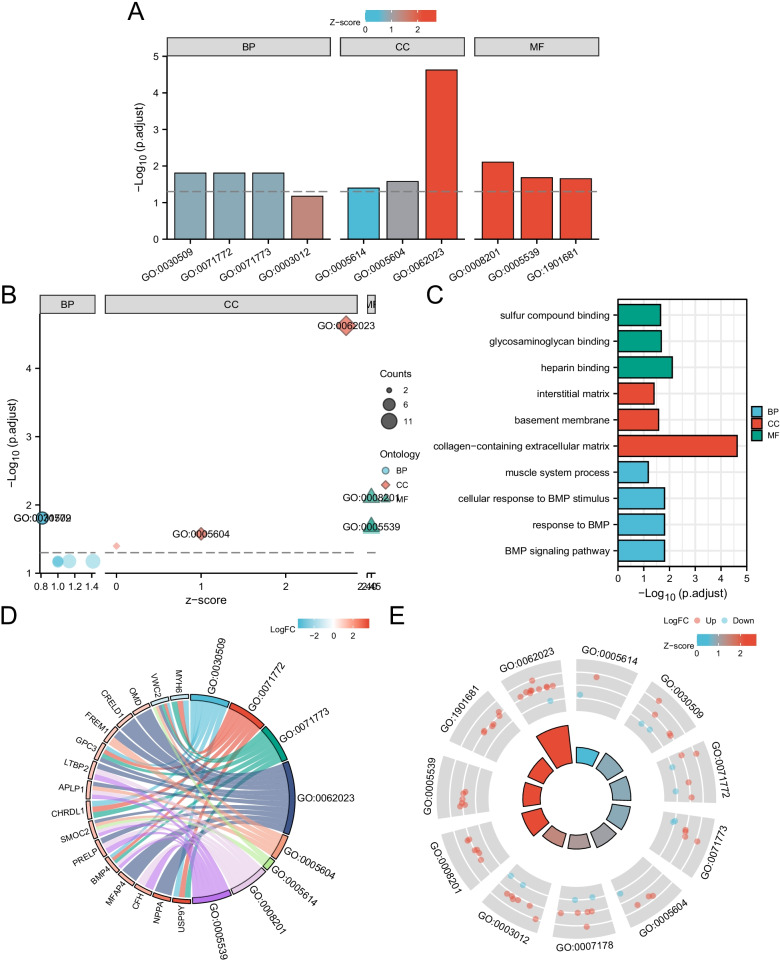
Enrichment plots by GO. (**A** GO Bar graph, **B** Bubble plot, **C** Bar graph of GO enrichment pathways, **D** chord diagram, **E** loop graph. BP: Biological Process; CC: Cellular Component; MF: Molecular Function)

In order to further study the biological functions of 10 DEGs, functional enrichment analysis was carried out, and the results are shown in Table 3. GO enrichment analysis showed that the functions of differentially expressed genes were mainly concentrated in the following 11 aspects: GO: 0030509 ~ BMP signaling pathway; GO: 0071772 ~ response to BMP; GO: 0071773 ~ cellular response to BMP stimulus; GO: 0003012 ~ muscle system process; GO: 0007178 ~ transmembrane receptor protein serine/threonine kinase signaling pathway; GO: 0062023 ~ collagen containing extracellular matrix; GO: 0005604 ~ basement membrane; GO: 0005614 ~ interstitial matrix; GO: 0008201 ~ heparin binding; GO: 0005539 ~ glycosaminoglycan binding; GO: 1901681 ~ sulfur compound binding. For the above GO meeting the requirements, the R language GOplot and ggplot 2 package are used for visual presentation (Fig. 4). According to the adjusted screening criteria of *P* value < 0.05 and Q value < 0.05, there was no enrichment pathway in KEGG.

**Table 3:** Significant enriched GO terms and pathways of DEGs

ONTOLOGY	ID	Description	GeneRatio	BgRatio	*p* value	p.adjust	q value
BP	GO:0030509	BMP signaling pathway	6/66	157/18670	1.93e−05	0.016	0.014
BP	GO:0071772	Response to BMP	6/66	170/18670	3.02e−05	0.016	0.014
BP	GO:0071773	Cellular response to BMP stimulus	6/66	170/18670	3.02e−05	0.016	0.014
BP	GO:0003012	Muscle system process	8/66	465/18670	2.26e−04	0.067	0.058
BP	GO:0007178	Transmembrane receptor protein serine/threonine kinase signaling pathway	7/66	349/18670	2.27e−04	0.067	0.058
CC	GO:0062023	Collagen-containing extracellular matrix	11/69	406/19717	1.54e−07	2.37e−05	2.06e−05
CC	GO:0005604	Basement membrane	4/69	95/19717	3.44e−04	0.026	0.023
CC	GO:0005614	Interstitial matrix	2/69	12/19717	7.79e−04	0.040	0.035
MF	GO:0008201	Heparin binding	6/66	169/17697	3.92e−05	0.008	0.007
MF	GO:0005539	Glycosaminoglycan binding	6/66	229/17697	2.09e−04	0.021	0.019
MF	GO:1901681	Sulfur compound binding	6/66	250/17697	3.35e−04	0.022	0.020

GO, Gene Ontology; DEGs, Differentially Expressed Genes

### GSEA enrichment analysis

**Fig. 5:**
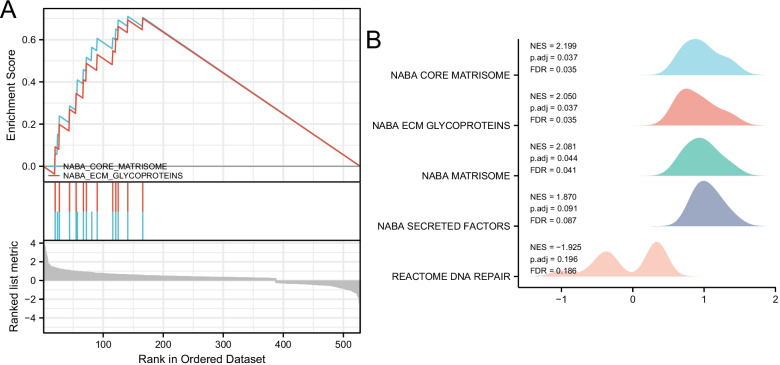
Enrichment plots by GSEA. (**A** GSEA Visual Analysis, **B** GSEA ridgeplot. NES: Normal Enrichment Score; FDR: False Discovery Rate)

GSEA was used to test the combined GEO dataset to identify functional gene sets associated with heart failure. Finally, five groups of HF related expressions were determined (Table 4), of which only three data sets met FDR Q value < 0.25 and *p*.adjust value < 0.05. These data sets include: (1) involved in encoding core extracellular matrix (including ECM glycoprotein, collagen and proteoglycan), (2) involved in encoding structural ECM glycoprotein, and (3) involved in encoding extracellular matrix and extracellular matrix related proteins (Fig. 3). According to the visualization results of GSEA, the eligible GSEA gene sets are as follows: (1) in NABA_ CORE_ Matrix gene set was significantly enriched (NES = 2.199; *p*.adjust = 0.037; FDR = 0.035); (2) At NABA_ ECM_ The glycoproteins gene set was significantly enriched (NES = 2.050; *p*.adjust = 0.037; FDR = 0.035) (Fig. 5).

**Table 4:** GSEA enrichment analysis

ID	Description	SetSize	EnrichmentScore	NES	*p* value	p.adjust	q values	Rank	Leading_edge
NABA_CORE_MATRISOME	Ensemble of genes encoding coreextracellular matrix including ECM glycol proteins, collagens and proteoglycans	15	0.7107363	2.199081	0.0014	0.03706	0.03519	141	Tags = 93%, List = 27%, Signal = 70%
NABA_ECM_GLYCOPROTEINS	Genes encoding structural ECM glycoproteins	12	0.7015504	2.049885	0.0015	0.03706	0.03519	166	Tags = 100%, List = 31%, Signal = 70%
NABA_MATRISOME	Ensemble of genes encoding extracellular matrix and extracellular matrix-associated proteins	44	0.5212222	2.08094	0.0026	0.04365	0.04144	141	Tags = 64%, List = 27%, Signal = 51%
NABA_SECRETED_FACTORS	Genes encoding secreted soluble factors	14	0.6127575	1.870019	0.0072	0.09133	0.08671	86	Tags = 64%, List = 16%, Signal = 55%
REACTOME_DNA_REPAIR	DNA Repair	13	0.543689	− 1.9251	0.0192	0.19553	0.18564	249	Tags = 100%, List = 47%, Signal = 54%

NES, Normalized Enrichment score

### Enrichment analysis by enrichr and reactome

**Fig. 6:**
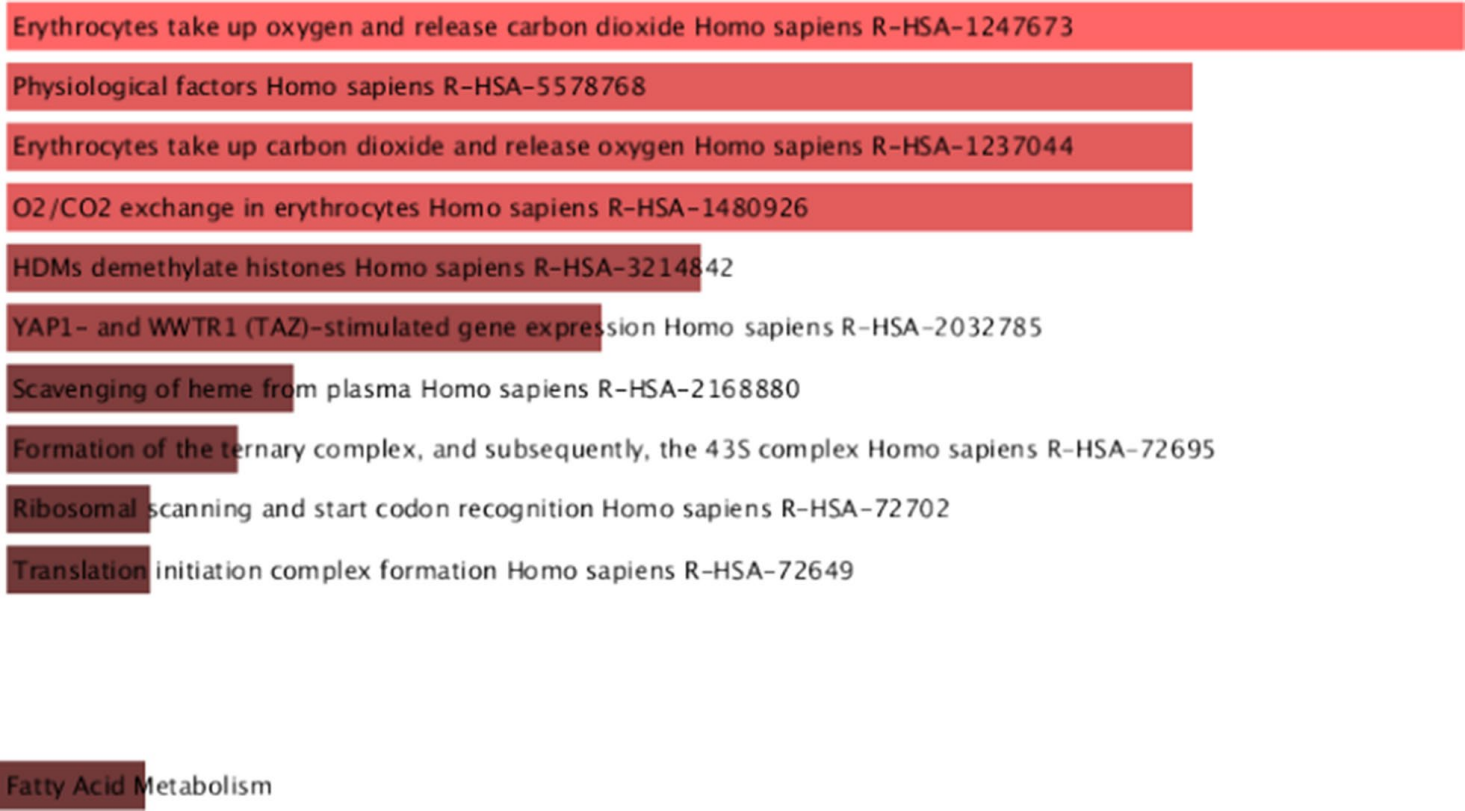
Enrichment pathways by Enrichr. (Upper: Related enrichment pathways of up-regulated genes; Lower: Related enrichment pathways of down-regulated genes)

Using Enrichr through online enrichment analysis, it was identified that 7 highly expressed IDS in HF group (EIF1AY, RPS4Y1, USP9Y, KDM5D, DDX3Y, NPPA and HBB) were related to Erythrocytes take up oxygen and release carbon dioxide, Physiological factors, Erythrocytes take up carbon dioxide and release oxygen, O_2_/CO_2_ exchange in erythrocytes, HDMs demethylate histones, YAP1-and WWTR1 (TAZ)-stimulated gene expression; 7 highly expressed IDs in normal group (TSIX, LOC28556 and XIST) were related to Fatty acid metabolism (Fig. 6). The ten DEGs identified were enriched and analyzed by Reactome database (https://reactome.org/), and the related biological processes of HF were not enriched.

### PPI network construction and hub gene selection

**Fig. 7:**
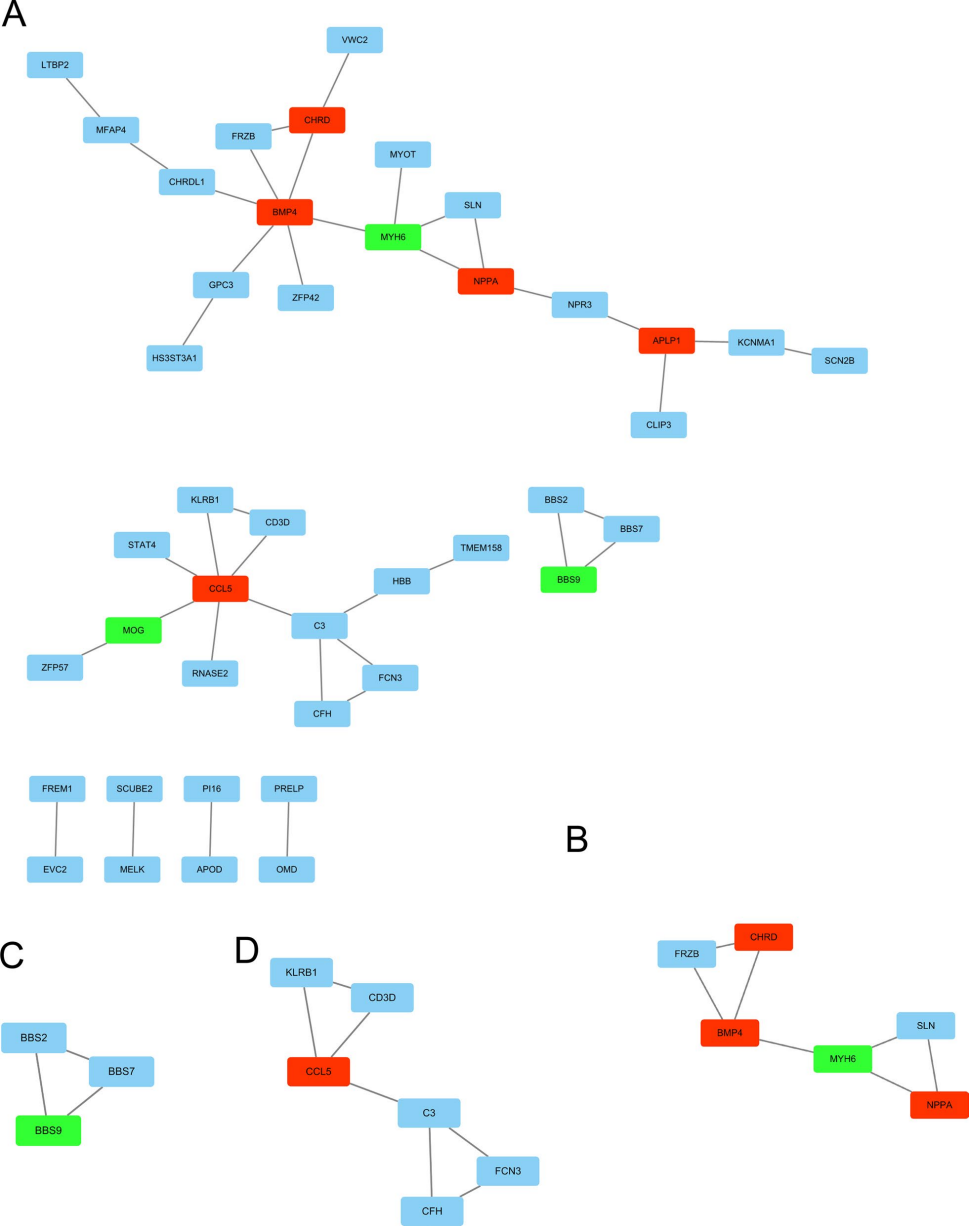
PPI network construction. (**A** The complete PPI network, **B**–**D** The three modules of hub genes)

PPI analysis was performed on these DEGs using the string platform, and 42 nodes and 41 interactions were finally determined (Fig. 7). In addition, an important module with 3 nodes and 3 edges is selected through MCODE. Two important modules with 6 nodes and 7 edges are selected through MCODE. BBS2, BBS7 and BBS9 are the hub nodes in module B, FRZB, CHRD, BMP4, MYH6, SLN and NPPA are the hub nodes in module C, and KLRB1, CD3D, CCL5, C3, CFH and FCN3 are the hub nodes in module D. Only BBS9, CHRD, BMP4, MYH6, NPPA and CCL5 were selected as hub genes. In addition, combined with the results of differential expression, enrichment analysis and PPI, BBS9, CHRD, BMP4, MYH6, NPPA and CCL5 were considered as Hub genes for further analysis.

### Identification of potential key genes associated with heart failure

**Fig. 8:**
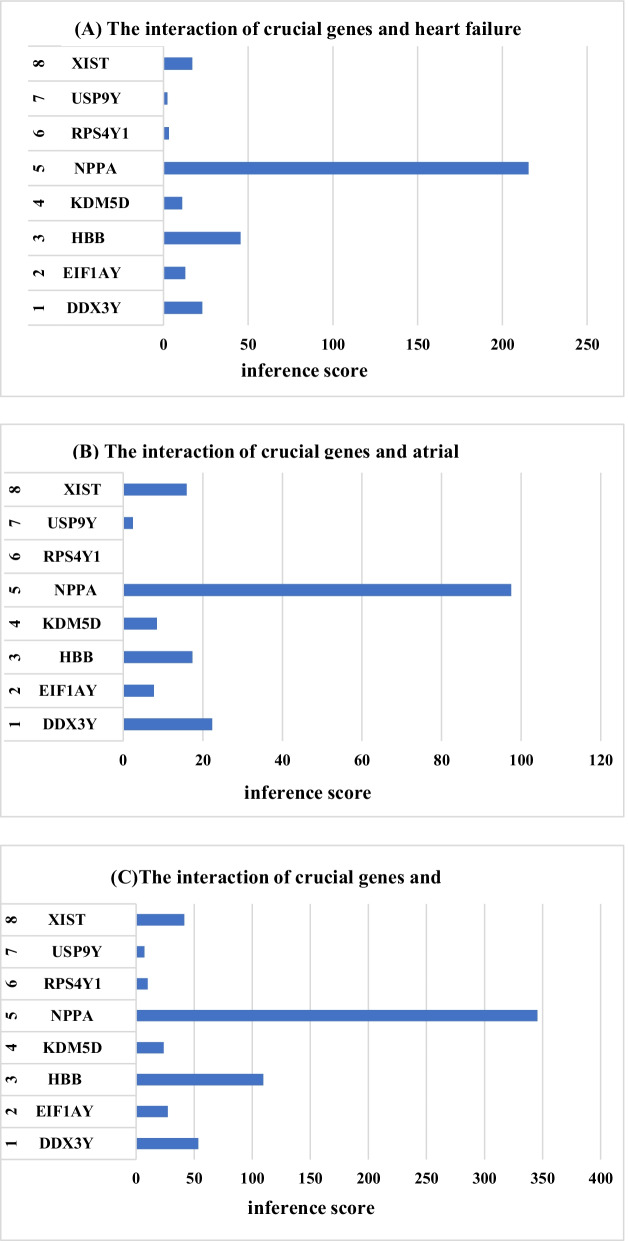

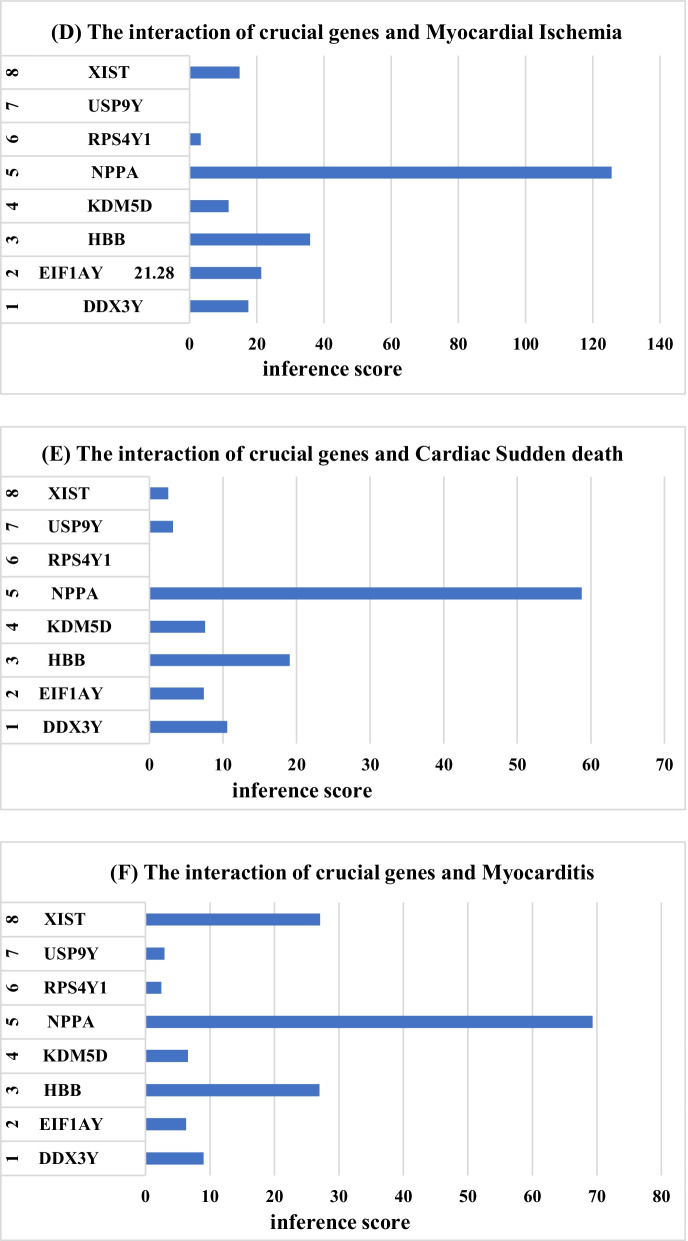

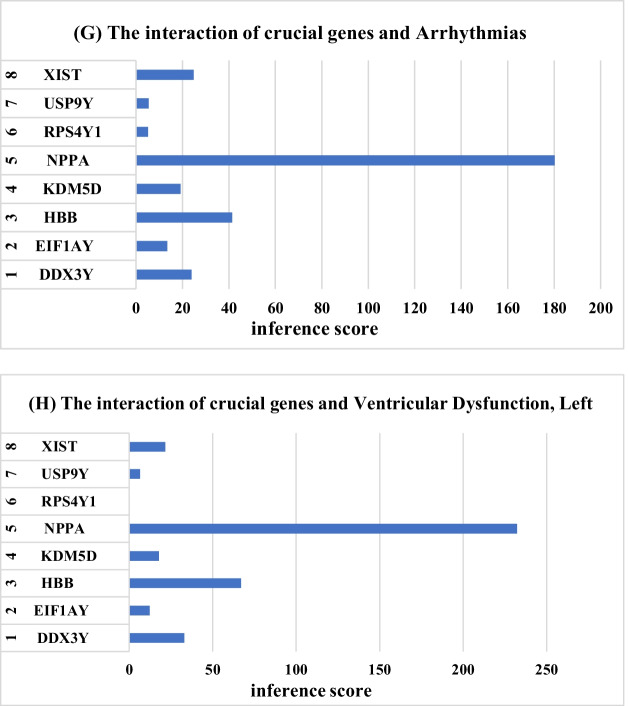
The CTD analysis between potential key genes and diseases

CTD is used to explore the interaction between potential key genes and heart failure. As shown in Fig. 8, potential key genes for heart failure, atrial fibrillation, hypertension, myocardial infarction, sudden cardiac death and myocarditis. The reasoning score in CTD reflects the association between chemicals, diseases and genes. The results of interaction showed that NPPA, HBB, DDX3Y and XIST had higher scores with heart failure.

## Discussion

At present, heart failure has made a great breakthrough in diagnosis, treatment and prevention compared with the past, and has achieved rich research results. In recent years, bioinformatics research such as biomarkers has made great progress and received more and more attention. Therefore, it is particularly important to study the biomarkers of heart failure for the diagnosis, treatment and prognosis of the disease. In this study, we integrated the gene expression profiles of 38 HF samples and 16 normal samples from 3 geo databases, and analyzed the data using bioinformatics tools. 85 IDs met the threshold of |log2 (FC)|≥ 1 & *p*.adj < 0.05. Under this threshold, 60 IDs were highly expressed in HF group and 25 IDS were highly expressed in normal group; 22 IDs met the threshold of |log2 (FC)|≥ 1.5 & *p*.adj < 0.05. Under this threshold, 16 IDs were highly expressed in HF group and 6 IDs were highly expressed in normal group; There are 10 IDs that meet the threshold of |log2 (FC)|≥ 2 & *p*.adj < 0.05. Under this threshold, there are 7 highly expressed IDs in HF group (EIF1AY, RPS4Y1, USP9Y, KDM5D, DDX3Y, NPPA, HBB) and 3 highly expressed IDs in normal group (TSIX, LOC28556, XIST). These 10 potential key genes (EIF1AY, RPS4Y1, USP9Y, KDM5D, DDX3Y, NPPA, HBB, TSIX, LOC28556, XIST) and some important pathways related to the risk of heart failure have been identified, indicating that these may play an important role in the mechanism of the occurrence and development of heart failure.

### Specific genes on the Y chromosome may be a risk factor for heart failure

EIF1AY, RPS4Y1, USP9Y, KDM5D [29] and DDX3Y, which are highly expressed in HF group, are located on Y chromosome. Considering that men account for a high proportion in the sample, such as data set GSE21610. However, gender is indeed a factor that cannot be ignored in cardiovascular diseases, especially in cardiovascular calcification [30]. In male sex hormones, there is a positive correlation between elevated testosterone and cardiovascular calcification, while in female sex hormones, the cardioprotective effect of estrogen is widely recognized. Therefore, when women enter menopause, the risk of cardiovascular disease will increase due to the decrease of estrogen level. When mineral components in the blood are deposited in blood vessels or heart valves, cardiovascular calcification can occur, including vascular calcification and heart valve calcification. According to its location, cardiovascular calcification can be divided into three types: atherosclerotic intimal vascular calcification, medial vascular calcification and aortic valve calcification [31]. Studies have shown that the occurrence of cardiovascular calcification has become a predictor of cardiovascular disease-related risks [32]. The deposition of maladjusted calcium may lead to coronary atherosclerotic heart disease, aortic stenosis, hypertension and even heart failure, and become the first trigger to push down dominoes. Studies have shown that in vascular smooth muscle cells, estrogen receptors α (ERα). The expression of estrogen receptor was higher than that of estrogen receptor β (ERβ). Estrogen is mainly through ERα Inhibit RANKL signal, so as to reduce osteogenic differentiation and calcification of vascular smooth muscle cells by up regulating BMP and down regulating MGP [33]. In addition, estrogen and aromatase in blood vessels may also play the same role [34]. On the other hand, studies have shown that matrix vesicles (MVS) produced by the outer membrane of cardiovascular cells It also plays a role in cardiovascular calcification. When they are ingested by vascular smooth muscle receptors, they can cause changes in MAPK signal and calcium metabolism. The regulatory response process of calcium binding annexin is activated, while the expression of calcification inhibitors such as MGP and fetuin A is at a low level [35, 36].

In conclusion, some key genes in this study may affect the cardiovascular system with the help of gender related factors, and finally play a promoting role in the occurrence and development of heart failure.

### Other highly expressed genes related to the mechanism of heart failure

The highly expressed NPPA gene encodes atrial natriuretic peptide (ANP) protein, which is involved in regulating humoral and electrolyte homeostasis together with BNP and CNP. ANP produces intracellular cGMP by binding to guanylate cyclase receptor on cell membrane, and binds to specific enzymes and ion channels, so as to play the biological function of natriuretic peptide [37, 38]. By studying the mixed muscle secretion phenotype of atrial cardiomyocytes, it is found that these cells can produce polypeptide hormone natriuretic peptide, so as to show the endocrine function of the heart. Studies have shown that NPPA is one of the earliest in situ gene expression during embryonic development, indicating that NPPA plays an important role in early cardiac development [39, 40]. Studies have shown that when acute heart failure occurs, the intermediate region sequence of pro—ANP (MR—proANP) may have greater advantages in judging the prognosis of patients [41]. In our results, compared with the normal group, NPPA was up-regulated in the heart tissue of heart failure, which was related to the traction stimulation of myocardium when heart failure occurred.

The highly expressed HBB gene encodes HBB protein, which is the key component of hemoglobin, so as to complete the important task of tissue oxygen supply. Studies have shown that patients with heart failure often have different degrees of hemodilution, and the changes of hemoglobin or hematocrit concentration can be used as an indirect marker to reflect congestion [42–45]. According to statistics, nearly one third of patients with heart failure are accompanied by anemia [46]. The existence of anemia may lead to more symptoms, increased hospitalization rate and increased mortality. Tissue and organ in patients with heart failure are often in an anoxic state. Studies show that erythropoietin levels in these patients are elevated and the overall concentration of erythropoietin is not significantly elevated due to retention of fluids [47, 48]. Erythropoietin is produced by the kidney and is the main stimulating factor for the production of red blood cells. Tissue hypoxia will promote the production of EPO, and the level of the latter is usually inversely proportional to the concentration of hemoglobin. Research shows that blocking β_2_-adrenergic receptors can reduce hemoglobin levels [49]. When heart failure occurs, sympathetic nerve excitability increases, which can promote the increase of hemoglobin level. Therefore, according to our results, compared with the normal group, HBB is highly expressed, which may be related to the abnormal activation of sympathetic nervous system and the level of erythropoietin in heart failure.

### Possible relationship between BMP, extracellular matrix and heart failure

Combining go enrichment analysis and PPI analysis, we pay attention to that BMP and extracellular matrix are relatively high-frequency keywords. Among them, XIST gene is also related to it.

Bone morphogenetic protein BMP is involved in the regulation of embryogenesis and organogenesis, and can participate in the development of cardiovascular structure and function in embryonic stage. When individuals mature, BMP can be used as an important endocrine regulator to participate in cardiovascular, metabolic and hematopoietic activities [50, 51]. Studies have shown that BMP is a transforming growth factor β (TGF-β), a member of the family carries out signal transmission with adjacent cells by means of paracrine or autocrine. Therefore, it plays a role in promoting the early development of organs. On the other hand, some BMP can also play a role in signal transmission in blood circulation, so as to affect distant tissues and organs, and finally complete the role of BMP in cardiovascular, metabolic and hematopoietic functions [52, 53]. Studies have shown that BMP signaling pathway plays an important role in cardiovascular diseases, increasing its activity in vascular inflammation and atherosclerosis, while decreasing its activity in pulmonary hypertension and hereditary hemorrhagic telangiectasia [53]. BMP has TGF-β, the commonness of the family is to bind to serine threonine kinase type I and type II receptors. Among them, the affinity for binding to type I receptors is higher, forming BMPRIA, also known as ALK3, BMPRIB, also known as ALK6, ACVRL1, also known as ALK1, and ACVR1, also known as ALK2 heterotetramer complexes; binding with type II receptors to form BMPR II, ACTRIIA, and ACTRIIB heterotetramer complexes, which can be widely expressed in mesenchymal stem cells In the tissues derived from, especially BMPR II is highly expressed in endothelial and endocardial tissues [54]. Studies have shown that when myocardial pathological hypertrophy is induced by pressure overload, the expression of BMP4 increases, but it does not increase in physiological hypertrophy induced by exercise [55, 56], and this effect can be inhibited by BMP inhibitors [56, 57]. BMP7 has been proved to inhibit cell apoptosis, myocardial fibrosis and anti calcification, and can improve the cardiac function of patients [58]. Studies have shown that BMP is closely related to the development of the heart during embryonic development. In the process of cardiac development, it needs to undergo endocardial to mesenchymal transition (EMT) And migrate as mesenchymal cells to fill the extracellular matrix separating the endocardial layer and the outer layer of myocardium [59, 60], and finally complete the development of the heart [61–63]. Therefore, the enriched pathways in this study are closely related to these mechanisms, which may provide some new evidence for these mechanisms [64].

Extracellular matrix (ECM) plays an important role in stabilizing the structure, transmitting signals and stress of cardiomyocytes, vascular cells and stromal cells. Therefore, the regulatory role of ECM is closely related to the occurrence and development of heart failure. The high expression of XIST gene in normal group is the main factor regulating the transcriptional silencing of X chromosome. At present, there are few studies on the role of XIST in cardiovascular diseases, mostly tumor related studies. It is reported that XIST and miR-101 can aggravate the occurrence of myocardial hypertrophy caused by excessive pressure load [65]. Studies have shown that silencing XIST can suck out mir-1277-5p through sponge action and inhibit the destruction of ECM [66, 67]. When cardiomyocytes are stimulated by external stimuli or their own dysfunction, it can affect the regulatory role of ECM and reduce stress The increase of load can cause the transformation of cardiac fibroblasts into myofibroblasts, that is, "interstitial fibrosis" And promote the synthesis of extracellular matrix, so as to reduce cardiac compliance, myocardial remodeling and accelerate the impairment of diastolic function [68]. Interstitial fibrosis is accompanied by the expansion of collagen area around cardiac microvascular adventitia, secondary to myocardial ischemia and hypoxia, aggravate the imbalance between supply and demand of blood oxygen under stress, and promote the occurrence and development of heart failure [69, 70]. Clinically, angiotensin converting enzyme inhibitors, angiotensin receptor inhibitors β [71, 72]. The application of adrenergic receptor antagonists and diuretics is to regulate the abnormal changes of ECM in patients with heart failure by reducing the load on the heart [73]. Among them, proteoglycan located in ECM plays a role in ECM, which belongs to glycosylated protein [74]. Research shows that fibroblasts' response to mechanical stress may include the following mechanisms: (1) After fibroblasts perceive the increase of mechanical load, they amplify the signal through intracellular cascade reaction and induce myocardial fibrosis by activating transcription factor myocardial related transcription factor (MRTF) [75]; (2) mechanical stress stimulates the key medium in the transformation of myofibroblasts—transforming growth factor TGF-β After treatment, it can promote matrix synthesis [76, 77]; (3) increased pressure load can directly activate renin angiotensin aldosterone system (RAAS), and stimulate fibroblast proliferation and ECM protein synthesis through angiotensin II type 1 receptor (AT1R) signal [78, 79]; (4) The increase of pressure negative charge can induce the expression of miRNA in fibroblasts, further activate MAPK signaling pathway, and finally promote the synthesis of matrix [80].

In the current research, the possible mechanisms of 10 potential key genes involved in the occurrence and development of heart failure have been discussed in the occurrence and development of heart failure. The results show that the above genes may become potential biomarkers and therapeutic targets of heart failure, hoping to provide some ideas for further exploration and research of heart failure Yes, this study still has some limitations: (1) the included samples have limitations: in the included data set, the age, gender, race, nationality, region, living habits and family history of the samples can be called influencing factors. (2) the potential key factors obtained from the analysis need to be experimentally verified in clinical samples, such as RT-qPCR, Western blot, etc.

## Conclusions

Our study integrated relatively large sample size data from multiple geographic data sets and identified 10 potential key genes (EIF1AY, RPS4Y1, USP9Y, KDM5D, DDX3Y, NPPA, HBB, TSIX, LOC28556, XIST) by bioinformatics analysis. The exploration of potential key genes of heart failure may provide some potential help for further identifying new biomarkers and useful therapeutic targets of heart failure susceptibility.

## Data Availability

The datasets analysed during the current study are available in the GEO Database repository, GSE76701 https://www.ncbi.nlm.nih.gov/geo/query/acc.cgi?acc=GSE76701. GSE21610 https://www.ncbi.nlm.nih.gov/geo/query/acc.cgi?acc=GSE21610. GSE8331 https://www.ncbi.nlm.nih.gov/geo/query/acc.cgi?acc=GSE8331.
